# Supplementary material for: Genome-Wide Comparison of the Target Genes of the Reactive Oxygen Species and Non-Reactive Oxygen Species Constituents of Cold Atmospheric Plasma in Cancer Cells
Source: Cancers (Basel). 2020 Sep 16;12(9):2640. doi: 10.3390/cancers12092640 (PMC7565996; doi:10.3390/cancers12092640)
Supplement: Supplementary file 1 [file cancers-12-02640-s001.pdf]

cancers

MDPI

Supplementary Materials

Genome-Wide Comparison of the Target Genes of the Reactive Oxygen Species and Non-Reactive Oxygen Species Constituents of Cold Atmospheric Plasma in Cancer Cells

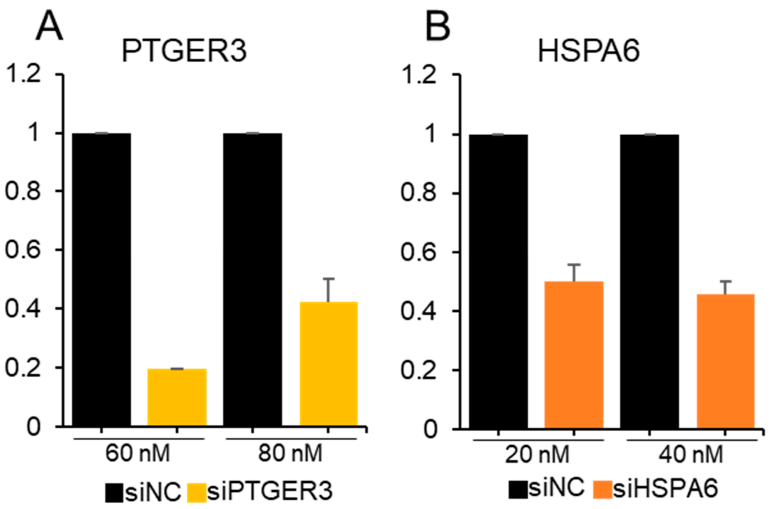

Figure S1. Induction of downregulation of PTGER3 and HSPA6 by a gene-specific siRNA in U937 cells. Cultured cells were transiently transfected with a gene-specific siRNA to downregulate PTGER3 (**A**) and HSPA6 (**B**) in U937 cells. Expression of the gene was determined by real-time RT-qPCR. Based on the downregulated level, 60 and 40 nM of siRNA were selected for PTGER3 and HSPA6, respectively. All experiments were performed in triplicate and indicated as mean ± SE. siNC, negative control siRNA; siPTGER3, siRNA for PTGER3; siHSPA6, siRNA for HSPA6.

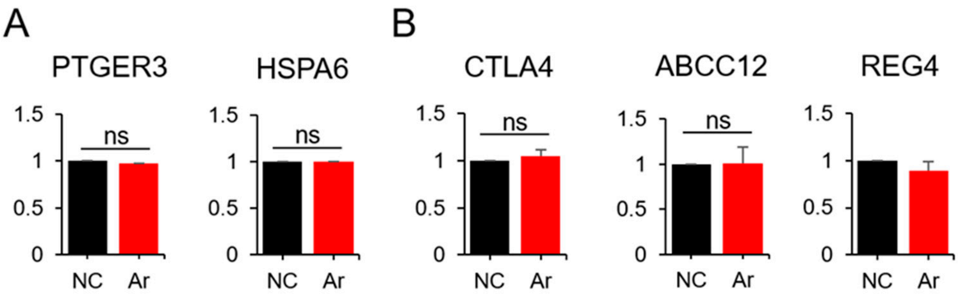

Figure S2. Effect of Ar gas on the expression of CAP- or H_2O_2-specific genes. To evaluate the effect of Ar gas alone on the expression of CAP- (**A**) or H_2O_2-specific genes (**B**), their expression was examined by RT-qPCR using RNA from the U937 cells treated with Ar gas or from non-treated cells. All assays were performed in triplicate, and the results are indicated as the mean ± SE. ns, non-significant.

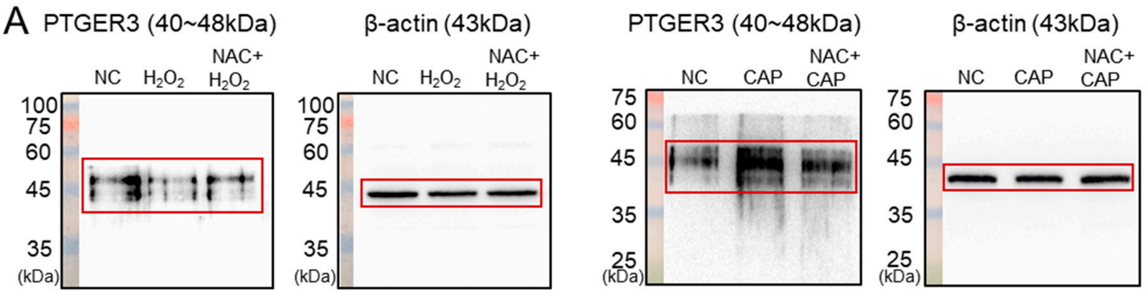

	NC	H2O2	NAC+H2O2
β-actin	14,857,988	10,726,528	11,582,608
PTGER3	12,501,740	8,251,956	9,144,112

	NC	CAP	NAC+CAP
β-actin	13,212,493	12,983,847	12,154,280
PTGER3	24,875,640	53,702,644	34,580,280

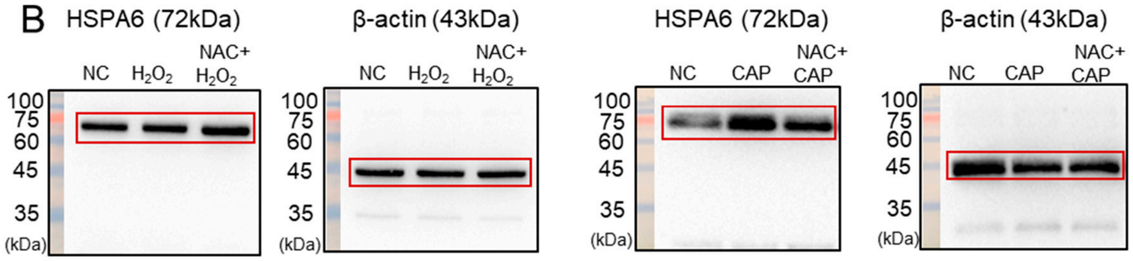

	NC	H2O2	NAC+H2O2
β-actin	8,561,148	6,270,296	6,533,752
PTGER3	2,186,628	4,312,776	3,281,424

	NC	CAP	NAC+CAP
β-actin	10,436,960	9,788,036	10,306,500
HSPA6	6,840,288	7,183,988	7,801,212

Figure S3. Uncropped blots corresponding to Western blots in the main text. Boxed areas correspond to the images in the main text. (**A**) Whole blots for Western blot analysis for Figure 5A. (**B**) Whole blots for Western blot analysis for Figure 5B.

Table S1. Information of primers for RT-qPCR and siRNAs used in this study.

Gene Symbol		Sequence (5′–3′)	Supplier
		qRT-PCR	
GAPDH	F:	ACATCGCTCAGACACCATG	IDT
	R:	TGTAGTTGAGGTCAATGAAGGG	
PTGER3	F:	GAGAGCAAGCGCAAGAAGTC	
	R:	CTTGGACAGGTACACGACGA	
HSPA6	F:	ACAGAGTGGCTGCCAAAAAC	
	R:	CCTGTCCTCTTCGGGAATCT	
CTLA4	F:	GAGTATGCATCTCCAGGCAAA	Bionics
	R:	TTCCCCATCATGTAGGTTGC	
ABCC12	F:	TGCCAAAAGATTTCGAGTCC	
	R:	ACGCGTGTCCTCTGGAATTT	
REG4	F:	TGTCCATCTTGTCGTCTTCG	
	R:	CACTGTCAGCTTCTGGGTTG	
		siRNA	
PTGER3	Sense:	GAAAGAUCCUUCUUCGAAAtt	Bioneer
	Antisense:	UUUCGAAGAAGGAUCUUUCtt	
HSPA6	Sense:	GUCUUCCAUGUGAAAGGUUtt	
	Antisense:	AACCUUUCACAUGGAAGACtt	
control		Negative control siRNA (SN-1003)	

Table S2. Genes with highly altered expression in U937 and SK-mel-147 cells after treatment with H_2O_2 and CAP.

Gene symbol	Accession No.	Description	U937		SK-mel-147		
			H_2O_2 [a]				
			4 h	18 h	3 h	6 h	24 h
REG4	NM_032044	regenerating family member 4	0.429	0.371	2.990	2.791	3.520
CTLA4	NM_005214	cytotoxic T-lymphocyte associated protein 4	0.275	0.225	3.124	4.992	4.604
MEG3	NR_003531	maternally expressed 3	0.283	0.328	2.409	2.783	2.918
ABCC12	NM_033226	ATP binding cassette subfamily C member12	2.462	4.959	3.023	2.963	4.412
			CAP				
			120 s_3 h [b]	120 s_3 h [c]	20 s_3 h [b]	60 s_3 h [b]	180 s_3 h [b]
PTGER3	NM_198717	prostaglandin E receptor 3	3.317	3.411	6.685	0.380	2.136
HSPA6	NM_002155	heat shock protein family A member 6	13.642	18.379	0.439	0.458	2.209

[a] The values are obtained by dividing the expression level of 3–24 h H_2O_2 treated cells by that of untreated cells. [b] The values are obtained by dividing the expression level of 20–180 s Ar-CAP treated and 3 h maintained cells by that of untreated cells. [c] The values are obtained by dividing the expression level of 120 s Ar+N_2-CAP treated and 3 h maintained cells by that of untreated cells.

© 2020 by the authors. Licensee MDPI, Basel, Switzerland. This article is an open access article distributed under the terms and conditions of the Creative Commons Attribution (CC BY) license (http://creativecommons.org/licenses/by/4.0/).
